# Prior to versus after Metformin Treatment—Effects on Steroid Enzymatic Activities

**DOI:** 10.3390/life13051094

**Published:** 2023-04-27

**Authors:** Benedikt Gasser, Genevieve Escher, Anca-Elena Calin, Michael Deppeler, Miriam Marchon, Hiten D. Mistry, Johann Kurz, Markus G. Mohaupt

**Affiliations:** 1 Department of Sport, Exercise and Health, Division Sport and Exercise Medicine, University of Basel, Grosse Allee 6, 4052 Basel, Switzerland; 2 Department of Biomedical Research, University Bern, 3006 Bern, Switzerland; 3 Lindenhofgruppe, Teaching Hospital of Internal Medicine, 3006 Berne, Switzerland; 4 Department of Women and Children’s Health, School of Life Course and Population Science, Kings College, London SE1 1UL, UK; hiten.mistry@kcl.ac.uk; 5 Interscience Research Collaboration, 8430 Leibnitz, Austria

**Keywords:** GC-MS, urine analysis, 11b-HSD deficiency, 21-hydroxylase, 17-hydroxylase, 11-hydroxylase, 3ß-HSD deficency

## Abstract

**Background**: We recently reported that metformin administration has substantial effects on steroid hormone concentrations. In this study, we specifically explored which enzymatic activities were affected before a first treatment versus after a time of metformin treatment. **Material and Methods:** Twelve male subjects (54.2 ± 9.1 years, 177.3 ± 4.1 cm, 80 ± 10.4 kg) and seven female subjects (57.2 ± 18.9 years, 162.7 ± 4.1 cm, 76.1 ± 10.4 kg) were recruited based on an indication of metformin. Prior to the first intake of metformin and after 24 h, urine collections were performed. Urine steroid analysis was completed using gas chromatography–mass spectrometry. **Results:** The average reduction in steroid hormone concentrations after the metformin treatment was substantial and relatively equally distributed in all metabolites and the sum of all metabolites with 35.4%. An exception was dehydroepiandrosterone, with a decrease of almost three hundred percent of average concentration. In addition, the sum of all cortisol metabolites and 18-OH cortisol (indicative of oxidative stress) were lower after the metformin treatment. Furthermore, significant inhibition of 3ß-HSD activity was detectable. **Discussion:** Effects prior to and after the metformin treatment on inhibiting 3ß-HSD activity were detected in line with findings from others. Furthermore, the pattern of a reduction, for example, in the sum of all glucocorticoids following the metformin treatment supported an effect on oxidative stress, which was further supported by the reduction in 18-OH cortisol. Nevertheless, we do not understand all steps in the complex pattern of the enzymes that affect steroid hormone metabolism and, consequently, further studies are necessary to improve our understanding.

## 1. Introduction

Despite the fact that metformin is an effective first-line treatment of type 2 diabetes mellitus and the most prescribed oral antiglycemic drug, its biochemical action is still not fully understood [1,2,3]. The primary effect of metformin is generally thought to be the inhibition of respiratory complex I (NADH: ubiquinone oxidoreductase) [4]. Verifying this, the inhibition of NADH-linked respiration by metformin was observed in mitochondria, submitochondrial particles [5] and immunoprecipitated complex I [6,7], which interact with the AMPK (AMP-activated protein kinase) system in cells [4]. However, no molecular-level or mechanistic understanding of the metformin–complex I interaction is currently known [4,8,9]. Besides targeting the abovementioned cascades, metformin was reported to inhibit several other enzymes linked to mitochondrial activity and steroid hormone metabolism [4,8,9]. It was recently reported that a dose-dependent effect on steroid hormone synthesis exists, with longer durations of metformin treatment resulting in more severe reductions of steroid hormone metabolites [10]. 

**Figure 1:**
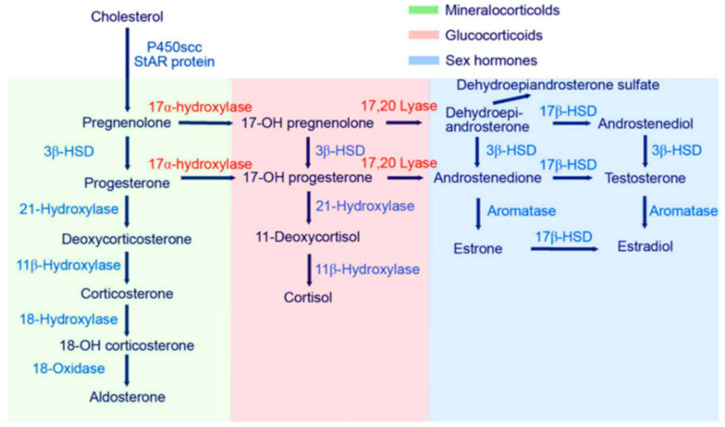
Steroidogenesis, starting with cholesterol and then routing to the different steroid hormones. Starting with cholesterol as a precursor of steroid metabolites, routing takes place in different organ systems (adrenal gland, sex organs, kidney and liver). Conversion from cholesterol to pregnenolone takes place in mitochondria through a P450 enzyme complex (hydroxylase/desmolase), which is induced through ACTH, linking the metabolite synthesis pathway with the HPA axis [24,25,26].

Steroid hormones mediate many cascades in the human body [10] (Figure 1); the dysregulation of these was identified in conditions such as high blood pressure, Cushing’s syndrome, post-traumatic stress disorders, depression and even autism [11,12]. Therefore, enzymes play a crucial role in targeting prenatal androgren exposure, such as aromatase or CYP17A1, and appear to influence mental health [13,14]. Studies on psychiatric morbidity among individuals with congenital adrenal hyperplasia (CAH), i.e., the inability to properly produce glucocorticoid, with increased androgen and dysregulated enzymatic activities were also reported [15]. Interestingly, individuals with 21-hydroxylase deficiency (one entity of CAH) have an increased risk of psychiatric disorders; this deficiency was also linked to autism [16,17]. Autistic traits were increased following prenatal exposure to abnormally high levels of testosterone caused by CAH, implying the higher the androgen concentration the more severe the disease [17]. Brain organization theory suggests that enzymatic activities, such as 21-hydroxylase, 17alpha hydroxylase or 3-beta hydroxysteroid, affect steroid hormones during fetal development, permanently organizing the brain for gender, including patterns of sexuality, cognition, temperament and interests [18]. This widely accepted theory has important implications for health, ranging from the medical management of infants with intersex conditions to suggested etiologies for sex differences in autism, depression and other mental health problems [18]. Studies of enzymatic alterations in females affected by different forms of CAH, in which high prenatal androgens were linked to both atypical genitals and “masculine” patterns of gender and sexuality, were performed [18]. To summarize, enzymes affecting steroid hormones influence many aspects of human health. Furthermore, the ratios of steroid hormones and, consequently, enzymatic activities have relevance for different diseases and influence the human phenotype [19]. For example, already in the 1970s, Margolese and Janiger used the ratios of androsterone and etiocholanolone to discriminate between heterosexual and homosexual phenotypes [19]. It was indicated that clinical diagnoses of Asperger’s and Kanner’s syndromes (DSM-IV) from the autistic spectrum were associated with strikingly different enzymatic activities, especially of 11-beta dehydrogenase, in the samples analyzed compared with their healthy matched controls by age, sex and BMI, implying that not only steroid hormone metabolites but also associated enzyme activities were affected [20]. It was also recently shown that enzymatic activity is dependent on oxidative stress, whereby a mediating effect via p38 was identified [21]. 

Metformin has only small reported side effects, such as nausea, which is troublesome but not necessarily dangerous, and lactic acidosis; however, this incidence is very low. Consequently, elucidating the effects of steroid hormones might be helpful to improve our understanding of the pharmacological targets [22]. The aim of this study was to first analyze enzymatic activities for both the glucocorticoid dysregulation 11b-HSD deficiency and 5a-reductase deficiency. Second, the activities indicating a potential CAH deficiency, such as 21-hydroxylase, 17-hydroxylase, 11-hydroxylase and 3ß-HSD deficiencies, and link these to oxidative stress via the ratio of 18-OH-cortisol to the sum of all cortisol metabolites. We hypothesized that a newly administered standard therapy with metformin has no effect on enzymatic activity in participants required to take this [23]. 

## 2. Materials and Methods

### 2.1. Participants

Thirteen male subjects (54.6 ± 9.0 years, 179.4 ± 4.2 cm, 84.4 ± 11.6 kg) and eight female subjects (55.1 ± 18.9 years, 162.5 ± 4.5 cm, 78.0 ± 10.0 kg) were recruited following written informed consent. Urine samples were collected as per the trial design below ready for analysis for steroid metabolites. Subjects were enrolled if they had an indication of Metformin based on the judgment of the general practitioner (GP), fulfilling the requirements as previously described [27]. Consequently, subjects needed to be older than 18 years and starting a new metformin treatment or were pausing treatment, for example, due to a further medical investigation for at least seven days. Excluded subjects were those suffering from a relevant disease, such as advanced renal failure, hepatic dysfunction, neoplasia or a musculoskeletal disease, as well as those with an active infection, under immunosuppressive therapy, under hormonal or steroid treatment, that were pregnant, that were known or suspected of malcompliance or drug or alcohol abuse.

### 2.2. Trial Design

During a consultation, the indication for metformin administration was given to the participants by their GP. Patients were asked if they were interested in participating and informed consent was given. Urine was collected for 24 h (24 h urine collections) before the first intake of metformin and a second time at the next appointment a few weeks later (for details, see the beginning of the Results section) after the treatment with metformin.

### 2.3. Steroid Measurement Procedures

Analysis of urinary steroids was conducted via gas chromatography–mass spectrometry (GC-MS) from urine collected before and after the metformin administration. The urine sample preparation comprised pre-extraction, enzymatic hydrolysis, extraction from the hydrolysis mixture, derivatization and gel filtration. The recovery standard was prepared by adding 2.5 µg of medroxyprogesterone to 1.5 mL of urine. The sample was extracted on a Sep-Pak C18 column (Waters Corp., Milford, MA, USA), dried, reconstituted in a 0.1 M acetate buffer (pH 4.6), and hydrolyzed with a powdered Helix pomatia enzyme (12.5 mg; Sigma Chemical Co., St. Louis, MI, USA) and 12.5 µL of β-glucuronidase/arylsulfatase liquid enzyme (Roche Diagnostics, Rotkreuz, Switzerland). The resulting free steroids were extracted on a Sep-Pak C18 cartridge. A mixture of internal standards (2.5 µg each of 5α-androstane-3α, 17α-diol, stigmasterol, cholesterol butyrate and 0.15 µg of 3β5β-tetrahydroaldosterone) was added to this extract, and the sample was derivatized to form the methyloxime-trimethylsilylethers. Analyses were performed on a Hewlett Packard gas chromatograph 6890 (Hewlett Packard, Palo Alto, CA, USA) with a mass selective detector 5973 via selective ion monitoring (SIM). One characteristic ion was chosen for each compound measured. The derivatized samples were analyzed during a temperature-programmed run (210–265 °C) over a 35 min period. The calibration standard consisted of a steroid mixture containing known quantities of all the steroid metabolites to be measured. Responses and retention times were recorded regularly. In each case, the ion peak was quantified against the internal stigmasterol standard. All procedures were performed as described several times by us and others [20,28,29,30,31]. 

### 2.4. Statistical Analyses

**Figure 2:**
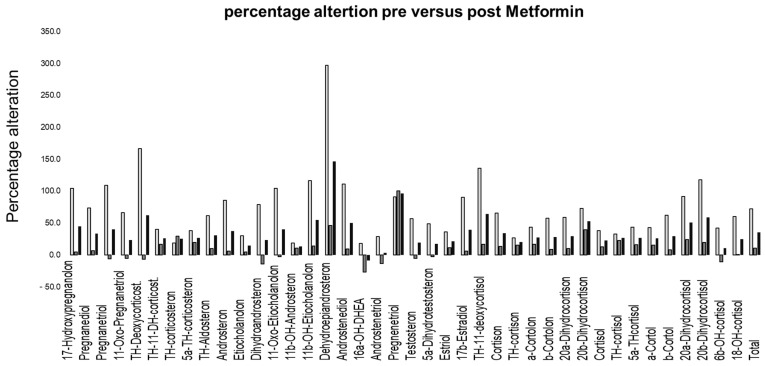
Percentage alterations for the reduction (*y*-axis) in the average concentration of metabolites pre- versus post-treatment with metformin (female light grey, male dark grey, total sample black).

The ratios of concentration levels of metabolites in 24 h urine indicative of enzymatic activities were also calculated (for abbreviations of the metabolites, see the legend of Table 1). For the 21-hydroxylase activity, the following ratios of metabolite concentrations were applied: 17HP/(THE+THFs), PT/(THE+THFs) and 100*PTONE/(THE+THFs). The following ratios indicative of 17-Hydroxylase activity were calculated: (THA+THBs)/(THE+THFs), (THA+THBs)/(AN+ET) and 100*THDOC/(THE+THFs). For 11-Hydroxylase activity, the following ratios were calculated: 100*THS/(THE+THFs) and 100*THDOC/(THE+THFs). For 3ß-HSD-activity: DHEA/(THE+THFs) and 5-PT/(THE+THFs); for 11b-HSD activity: Cortisol/Cortison, (THFs)/THE and (F+E)/(THE+THFs); and for 5a-reductase-deficiency: THF/5aTHF. For all ratios, the mean and SD for the females, males and total sample were calculated. To analyze a potential normal distribution of the measured values of each metabolite, Jarque–Bera tests were performed. As in most cases of metabolite concentrations, for all subsamples (female, male, total sample), the normal distribution was identified and differences between pre- versus post-intervention were analyzed with two-tailed paired *t*-tests. Percentage alterations between measurements from 24 h urine concentration levels of each metabolite and for the sum of all metabolites were calculated regarding before versus after metformin treatment (Figure 2). A linear regression model (ordinary least squares) for the relationship between creatinine excretion and the sum of all metabolites for the total sample, along with calculating the coefficient of determination (R^2^), was performed. All calculations were performed with Microsoft Excel (Microsoft Inc., Redmond, WA, USA).

## 3. Results

**Figure 3:**
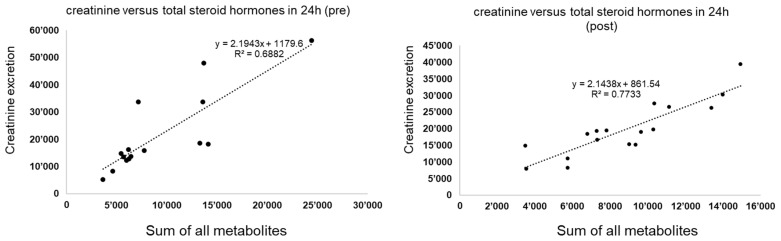
The general linear relationship between the sum of all steroid hormones (*x*-axis) and the creatinine excretion (*y*-axis) before the metformin treatment (**left**) and after the metformin treatment (**right**) for the total sample. The patterns before versus after are almost the same.

The average daily intake was 0.9 ± 0.1 g of metformin for the males and 0.97 ± 0.1 g of metformin for the females, whereby no significant difference in average daily intake was detected between male versus female subjects (*p* = 0.73). The average treatment duration between the pre-test and post-test was 47 ± 16.6 days for the males and 42.4 ± 15.4 for the females (*p* = 0.88). To the best of our knowledge, no relevant endocrine disorders were present in the male participants. Of the females, two had polycystic ovary syndrome (PCOS), though it was a very mild form in one of the women. Figure 2 gives an overview of the percentage alteration for each metabolite, before and after the metformin administration. Despite a substantially higher reduction in dehydroepiandrosterone post-metformin, most other metabolites showed a reduction within the same range (large reference intervals are described, see Ackermann et al. [32]). The average reduction in concentration levels in the females was 72%, 11% in the males and 35% for the total sample, with the effects of metformin being significant in the females (*p* < 0.01). Figure 3 shows the general linear relationship between the sum of all steroid hormones and the creatinine excretion in the urine. In both situations, i.e., before and after metformin administration, an almost identical linear relationship was detected despite the fact that the subjects were not specifically controlled for factors that affect creatinine excretion (e.g., meat consumption or physical activity). 

Table 1 gives an overview of the calculated ratios of the metabolites indicative of 21-hydroxylase, 17-hydroxylase, 11-hydroxylase and 3-beta hydroxysteroid activities, as well as 11-beta hydroxy steroid and 5-alpha reductase deficiencies, showing no specific alterations for the entire together. However, the sum of all cortisol metabolites was higher before versus after the metformin treatment. In addition, 18-OH cortisol was lower after the metformin administration.

## 4. Discussion

The aim of this study was to elucidate the influences of metformin on enzymatic activities in depth. The average treatment duration in the analyzed samples was around 6–7 weeks. In principle, saturated levels should have been achieved after 5 half-life times; however, the physiological effect on the human body takes more time [22]. The elimination half-life time of metformin during multiple dosages in patients with good renal function is approximately 5 h, and thus, the treatment duration of around 6–7 weeks was sufficient to provide saturated levels and presumably the respective physiological effects as previously described [1,2,3,4,5,6,7,8,9,22]. Good renal function was assumed for the analyzed samples, as these were individuals with a new indication for metformin prescription and not having already developed conditions such as diabetic nephropathy [33]. This was further supported by the fact that creatinine excretion and the sum of all measured steroid hormones showed a highly linear dependency with high coefficients of determination both before and after the metformin administration for the total of all samples, in line with the general understanding of a higher excretion of steroid hormones with a higher renal activity (see Figure 3) [20,28,29,30,31]. 

Chemically, metformin is a hydrophilic base that exists at physiological pH as the cationic species (>99.9%) [22]. Consequently, its passive diffusion through cell membranes is potentially very limited, explaining no differences in 17-alpha hydroxylase [22]. The oral absorption, hepatic uptake and renal excretion of metformin are greatly mediated by organic cation transporters (OCTs), which were not measured in this study, and thus, a limitation of this study. Nevertheless, the effects of structural variants of OCTs and other cation transporters on the pharmacokinetics of metformin appear to be small and the subsequent effects on the clinical response are limited [22]. However, intersubjective differences in the levels of expression of OCT1 and OCT3 in the liver are very large and might contribute more to the variations in the hepatic uptake and clinical effect of metformin [22]. In principle, diagnostic ratios calculated from urinary steroid hormone metabolites can be used as a measure for the relative activity of steroidogenic enzymes or pathways in the clinical investigation of steroid metabolism disorders [34]. As such, age-specific effects on steroid hormone excretion were described, yielding another limitation of this study, as a relatively broad age range was analyzed. Ideally, all subjects analyzed would have the same age and other confounding factors, such as daily stress levels; hours of sleep; and nutritional intake, such as meat consumed, held constant. 

It was previously reported that metformin had serious effects on steroid hormone levels [10]. We found that enzymatic activities were partly altered after the metformin treatment. Significant effects for all samples analyzed were found in the activity of 3ß-HSD, the sum of all cortisol metabolites and 18-OH cortisol. The roles of 5α reductase and 11-b-hydroxysteroid deficiencies, as well as 11-hydroxylase, on 21-hydroxylase are ambiguous, and 17-hydroxylase seems to have no significant effect. The average reduction in steroid hormone concentrations was substantial and relatively equally distributed in all metabolites and the sum of all metabolites. The exception was dehydroepiandrosterone, with a higher reduction, in line with the detected alteration of 3ß-HSD (Figure 2). The large ranges of concentrations considered normal have to be kept in mind (discussed in Ackermann et al. [32]). The average reduction in the total sample of steroid hormone concentrations was 35.4 percent, which might be valid (Figure 1). Focusing on the details, the large effect on dehydroepiandrosterone was in line with the alteration of 17,20 Lyase activity but not necessarily that of 17-alpha Hydroxylase [13]. As no significant effect on 17-alpha hydroxylase was detected, a role of oxidative stress was implied, which uniquely mediated 17,20 Lyase activity but not 17-alpha hydroxylase activity, as previously reported [13,21,35]. 17,20 Lyase activity catalyzes the activity of adrenal P450c17 promoted by Mitogen-activated protein kinase 14 (MAPK14, p38α) [13,21,35]. This is the kinase responsible for enhancing 17,20-lyase activity through P450c17 phosphorylation [13,21,35]. Through its oxidant-sensitive activity, an increase in 17.20-lyase activity mediated by p38α occurs under oxidative stress [21]. Furthermore, the alterations in the sum of all glucocorticoids and reduction in 18-OH cortisol are indicative of the influence of metformin on oxidative stress. Hirsch et al. showed at the cell level that metformin modulates androgen production in NCI-H295R cells (an established model of steroidogenesis) [36]. Similar to the in vivo situation, metformin inhibits androgen production in NCI cells by decreasing the activity of 3ß-hydroxysteroid and CYP17A1 (not detected in our study) [36]. Our findings concur with those by Vrbikova et al. in 2001, who analyzed 24 women with PCOS before and after treatment (27 ± 4 weeks) with metformin (1 g/day) [37]. The treatment duration was longer for males (males 47 ± 16.6 days, females 42.4 ± 15.4 days), but the dosage was similar (average daily intake in our sample was 0.9 ± 0.1 g for males and 0.97 ± 0.1 g for females). They detected no significant change in basal plasma steroid levels [37]. After ACTH stimulation (not performed in our study), significant decreases in the activities of 3 beta-hydroxysteroid dehydrogenase and 17 beta-hydroxysteroid dehydrogenase were observed, as well as an increase in the activity of C17,20-lyase [37]. Similar to our findings, a significant decrease in 3 beta-hydroxysteroid dehydrogenase was detected [37]. Differences in the findings might have been due to the measurements in plasma versus urine here, whereby, for example, UPD polymorphism plays a relevant role [38]. Furthermore, differences might have been due to the longer treatment duration yielding a steady state, which reduced the effect of metformin. 

To conclude, steroidogenesis was affected by metformin in this study, which consisted mainly of healthy subjects with the exception of metformin indication, due to increased blood sugar levels. Steroidogenesis starts with cholesterol and then routing is affected by the enzymatic activities of the analyzed enzymes ranging from acetate to cholesterol as precursors of steroid hormone metabolites. Therefore, routing takes place in different organs (adrenal gland, sex organs, kidney and liver), whereby conversion from cholesterol to pregnenolone takes place in mitochondria through a P450 enzyme complex (hydroxylase/desmolase), which is induced through ACTH, linking the metabolite synthesis pathway with the hypothalamus pituitary adrenal axis [24,25,26]. Consequently, nearly all steroid hormone concentrations were lower after treatment with metformin. It is likely that synthesis per se was affected, and not only enzymatic activity, as shown here with, for example, 3ß-HSD activity. This is in line with other findings that showed the effects of metformin treatment on reduced low-density lipoprotein and cholesterol levels [39] at the cellular level, where it was directly indicated that metformin inhibited cholesterol synthesis [40]. Thus, an effect of metformin on the level of the precursor cholesterol was already described. To summarize, the effects of metformin on 3ß-HSD activity, glucocorticoid activity and oxidative stress were identified. Metformin appears to be a safe pharmaceutical agent that might be considered for off-label uses at targeting the respective pathways due to its safety and considering the small side effects.

## 5. Highlights

Metformin is an effective first-line treatment of type 2 diabetes mellitus and the most prescribed oral antiglycemic drug; however, its biochemical action is still not fully understood. Furthermore, Metformin is the treatment of choice for the metabolic consequences seen in polycystic ovary syndrome due to its insulin-sensitizing and androgen-lowering properties.

Therefore, we collected 24 h urine in female and male subjects with an indication for metformin twice, namely, before and after Metformin intake, and performed an encompassing steroid hormone analysis with gas chromatography–mass spectrometry (GC-MS). 

We detected the effect of metformin on enzymes influencing steroidogenesis in female and male subjects with an indication for metformin before the first intake of metformin versus after approximately six weeks of metformin intake of a standard dosage of around 1 g per day

Most pronounced were the effects on the metabolite Dehydroepiandosterone (DHEA), which showed a substantial decrease after administration of Metformin. 

This implies an effect on the 3ß-HSD activity (Hydroxysteroid dehydrogenase) based on the calculated indices. As the sum of all cortisol metabolites and 18-OH cortisol were lower after the metformin treatment, an effect on oxidative stress was implied

No association before versus after the intake of metformin and creatinine clearance was detected in the total sample of female and male subjects; furthermore, no significant effect was detected on the enzymatic activities of 21-hydroxylase, 17-hydroxylase, 11-hydroxylase, 11-beta hydroxy steroid and 5-alpha reductase in the total sample of female and male subjects.

Interestingly, the findings of the inhibition of 3ß-HSD activity are in line with the ones recently published by Hirsch et al., which showed that metformin modulates androgen production in NCI-H295R cells (an established model of steroidogenesis). Similar to in vivo situations and the findings in our study, metformin inhibited androgen production in NCI cells by decreasing the HSD3B2 expression. The effect of metformin on androgen production was dose-dependent and subject to the presence of organic cation transporters, establishing an important role of organic cation transporters for metformin’s action in line with our findings on the markers of oxidative stress. The findings here and the ones by Hirsch et al. imply an effect on mitochondrial complex I, which was already described as the main target of metformin action.

Nevertheless, the complex action of Metformin is still not finally understood, as we do not know all the cascades and steps targeted. The complex pattern of enzymes affecting steroid hormone metabolism makes an intuitive and/or mathematical understanding impossible and so far only allows for analysis at the descriptive level.

## Figures and Tables

**Table 1:** Enzyme activities for the female sample (n = 8), male sample (n = 13) and total sample (n = 21). Data are given as ratios of metabolites. Abbreviations used are as follows: THA for Tetrahydrodehydrocorticosteron, THB for Tetrahydrocorticosteron, 5aTHB for 5a-Tetrahydrocorticosteron, THS for TH-11-deoxycortisol, HP for 17-Hydroxypregnalonon, PT for Pregnanetriol, PT’ONE for 11-Oxo-Pregnanetriol, THDOC for Tetrahydrodoc, E for Cortison, THE for Tetrahydrocortison, F for Cortisol, THF for Tetrahydrocortisol, 5ATHF for 5-alphaTetrahydrocortisol and 18-OH-F for 18-OH-cortisol. Color-coded *p*-values: red > 0.5, light green 0.1 < *p* < 0.5 and *p* < 0.1 dark green.

Ratios Indicative for Enzymatic Activity	Total Sample (Pre versus Post)					Female Sample (Pre versus Post)					Male Sample (Pre versus Post)				
	Mean	SD	*p*-Value	Mean	SD	Mean	SD	*p*-Value	Mean	SD	Mean	SD	*p*-Value	Mean	SD
**21-Hydroxylase activity**															
17HP/(THE+THFs)	0.028	0.030	0.836	0.024	0.031	0.026	0.011	0.910	0.028	0.028	0.029	0.011	0.778	0.030	0.011
PT/(THE+THFs)	0.115	0.155	0.532	0.100	0.117	0.113	0.062	0.779	0.125	0.095	0.109	0.078	0.274	0.117	0.070
100*PTONE/(THE+THFs)	0.289	0.604	0.253	0.698	2.015	0.227	0.091	0.376	0.825	1.382	0.219	0.135	0.209	0.293	0.167
**17-Hydroxylase activity**															
(THA+THBs)/(THE+THFs)	0.050	0.060	0.872	0.043	0.036	0.070	0.031	0.836	0.072	0.028	0.045	0.016	0.609	0.043	0.014
(THA+THBs)/(AN+ET)	0.079	0.075	0.674	0.073	0.046	0.188	0.124	0.856	0.192	0.140	0.101	0.099	0.486	0.092	0.085
100*THDOC/(THE+THFs)	0.260	0.531	0.703	0.204	0.160	0.335	0.333	0.603	0.247	0.200	0.199	0.061	0.059	0.297	0.121
**11-Hydroxylase activity**															
100*THS/(THE+THFs)	1.562	1.422	0.813	1.486	1.314	1.742	0.746	0.396	1.397	0.433	1.631	0.600	0.194	1.783	0.854
100*THDOC/(THE+THFs)	0.260	0.531	0.703	0.204	0.160	0.335	0.333	0.603	0.247	0.200	0.199	0.061	0.059	0.297	0.121
**3ß-HSD activity**															
DHEA/(THE+THFs)	0.136	0.419	0.218	0.054	0.121	0.137	0.289	0.358	0.039	0.078	0.073	0.101	0.160	0.058	0.089
5-PT/(THE+THFs)	0.023	0.046	0.009	0.014	0.023	0.025	0.028	0.027	0.017	0.024	0.016	0.017	0.111	0.010	0.013
**GSH**															
18-OH-F	192.628	40.363	0.093	161.085	30.806	404.673	157.727	0.105	201.125	97.415	156.722	79.921	0.663	147.504	79.674
18-OH-F/Cortisolmetabolites	0.014	0.013	0.900	0.016	0.014	0.026	0.007	0.374	0.022	0.005	0.015	0.008	0.208	0.016	0.007
**Glucocorticoides**															
Sum of all Glucocortiocoids	14163.684	3184.700	0.015	10305.908	2205.948	15131.494	3010.560	0.095	9000.381	3771.801	12700.851	9741.524	0.111	9884.749	6381.372
**11b-HSD deficiency**															
Cortisol/Cortison	2.165	2.849	0.585	2.093	2.195	2.564	2.344	0.621	2.139	0.733	2.090	0.720	0.881	2.042	0.740
(THFs)/THE	0.488	0.314	0.963	0.535	0.407	0.511	0.110	0.669	0.563	0.216	0.632	0.199	0.499	0.603	0.187
(F+E)/(THE+THFs)	0.085	0.093	0.903	0.076	0.080	0.085	0.024	0.332	0.067	0.022	0.092	0.032	0.172	0.102	0.037
**5a-reductase deficiency**															
THF/5aTHF	16.746	17.749	0.635	17.817	17.837	14.773	2.226	0.208	20.493	9.924	15.599	5.803	0.065	13.488	4.580

## Data Availability

Data is available on qualified request to the corresponding author.
